# Emergency Nurses’ Reasons for Not Recommending Their Hospital to Clinicians as a Good Place to Work

**DOI:** 10.1001/jamanetworkopen.2024.4087

**Published:** 2024-04-09

**Authors:** K. Jane Muir, Raina M. Merchant, Karen B. Lasater, J. Margo Brooks Carthon

**Affiliations:** 1 Center for Health Outcomes and Policy Research, School of Nursing, University of Pennsylvania, Philadelphia; 2 The Leonard Davis Institute of Health Economics, University of Pennsylvania, Philadelphia; 3 National Clinician Scholars Program, University of Pennsylvania, Philadelphia; 4 Department of Emergency Medicine, University of Pennsylvania, Philadelphia

## Abstract

**Importance:**

Half of emergency nurses report high burnout and intend to leave their job in the next year. Whether emergency nurses would recommend their workplace to other clinicians may be an important indicator of a hospital’s ability to recruit clinicians.

**Objective:**

To examine why emergency nurses do not recommend their hospital to other clinicians as a good place to work.

**Design, Setting, and Participants:**

This qualitative study used directed content analysis of open-text responses (n = 142) from the RN4CAST-NY/IL survey of registered nurses licensed in New York and Illinois between April 13 and June 22, 2021. Inductive and deductive analytic approaches guided study theme development informed by the Social Ecological Model. The collected data were analyzed from April to June 2023.

**Main Outcomes and Measures:**

Nurses who answered “probably not” or “definitely not” to the survey question, “Would you recommend your place of employment as a good place to work?” were prompted to provide a rationale in an open-text response.

**Results:**

In this qualitative study of 142 emergency nurses (mean [SD] age, 43.5 [12.5] years; 113 [79.6%] female; mean [SD] experience, 14.0 [12.2] years), 94 (66.2%) were licensed to work in New York and the other 48 (33.8%) in Illinois. Five themes and associated subthemes emerged from the data. Themes conveyed understaffing of nurses and ancillary support (theme 1: unlimited patients with limited support); inadequate responsiveness from unit management to work environment safety concerns (theme 2: unanswered calls for help); perceptions that nurses’ licenses were in jeopardy given unsafe working conditions and compromised care quality (theme 3: license always on the line); workplace violence on a patient-to-nurse, clinician-to-nurse, and systems level (theme 4: multidimensional workplace violence); and nurse reports of being undervalued by hospital management and unfulfilled at work in delivering suboptimal care to patients in unsafe working conditions (theme 5: undervalued and unfulfilled).

**Conclusions and Relevance:**

This study found that emergency department nurses did not recommend their workplace to other clinicians as a good place to work because of poor nurse and ancillary staffing, nonresponsive hospital leadership, unsafe working conditions, workplace violence, and a lack of feeling valued. These findings inform aspects of the work environment that employers can address to improve nurse recruitment and retention.

## Key Points

**Question:**

Why do emergency nurses not recommend their hospital to clinicians as a good place to work?

**Findings:**

This qualitative study was a directed content analysis of 142 open-text responses from emergency nurses describing why they indicated “probably not” or “definitely not” to the survey question, “Would you recommend your place of employment as a good place to work?” Nurses reported insufficient staffing, nonresponsive unit administration, unsafe working conditions, workplace violence, and a sense of being undervalued as reasons for not recommending their workplace to clinicians.

**Meaning:**

These findings provide opportunities for health care administrators to improve emergency departments for clinician retention.

## Teaser

This qualitative study investigates responses from registered nurses employed in hospital emergency departments in New York and Illinois about the reasons they would not recommend their workplace to other clinicians.

## Introduction

Mounting evidence on the quality of emergency department (ED) work environments^1^ and clinician job outcomes^1,2,3^ suggests that hospital EDs are a less appealing workplace for clinicians compared with other acute care settings. Approximately 1 in 3 emergency nurses are dissatisfied with their jobs, 53% report high burnout, and almost half intend to leave their job in the next year.^1,2^ On average, emergency nurses provide significantly worse ratings of their work environments and staffing levels compared with their inpatient unit colleagues.^2^ Other evidence demonstrates an association between poor ED work environments and emergency nurse intent to leave the job.^1^ In the context of attracting nurses to fill hospital vacancies,^4^ it is unknown why emergency nurses would not recommend their workplace to other clinicians. As employers struggle to recruit and retain nurses across all care settings, there is a strong impetus to identify retention strategies for emergency nurses given that nearly 50%^5^ of hospital-based care is initiated in the ED.

This study investigates responses from registered nurses employed in hospital EDs in New York and Illinois about the reasons they would not recommend their workplace to other clinicians. Findings provide actionable information for hospital administrators to support ED nurses’ work and inform interventions that may improve attraction and retention of ED nurses.

## Methods

### Data Source

This qualitative, descriptive study is a directed content analysis of open-text responses from registered nurses working as direct care staff nurses in a New York or Illinois hospital ED between April 13 and June 22, 2021. The purpose of directed content analysis is to focus on the characteristics of language as communication to the content or context meaning of text.^6,7,8^ Nurse responses were obtained via the RN4CAST NY/IL^9^ survey, which was a large email-based survey conducted to evaluate nurse demographics, nurse job outcomes, and the quality of nurses’ work environments by sampling 100% of licensed nurses in the study states. Survey questions were multiple choice, select all that apply, and open-text response. This study received approval from the institutional review board at the University of Pennsylvania. Participants provided written informed consent before initiating the survey. The Consolidated Criteria for Reporting Qualitative Research (COREQ) reporting guidelines for qualitative studies were followed.

Germane to this study, nurses were asked about their setting of employment, position, and responded to the question, “Would you recommend your place of employment as a good place to work?” The responses to this question were reported on a Likert scale from 1 (definitely yes) to 4 (definitely not). Nurses who indicated “probably not” or “definitely not” were prompted to complete the open-ended response describing why they would not recommend their hospital.

Among the 1526 emergency nurses who answered the question about recommending their workplace, 621 nurses (40.7%) indicated that they would not recommend their workplace, and 142 of these nurses (22.8%) provided a rationale via the open-text response. Participants also provided demographic information (race, ethnicity, age, sex, and years of work experience). Race and ethnicity were collected in the study to determine the variation in emergency nurse reports on their workplace by demographic characteristics. Reported racial categories included American Indian or Native Hawaiian, Asian, Black or African American, White, multiracial, and other (no additional information available); however, numbers could not be reported for categories except White due to small cell sizes. In addition, the ethnic category of non-Hispanic could not be reported due to small cell size.

### Conceptual Framework

The conceptual framework guiding data analysis was the Social Ecological Model,^10,11,12^ which posits that studied phenomena can influence and impact individuals, interpersonal dynamics, and larger systems. These levels are embedded and influence one another. Applied to the current study, the Social Ecological Model situates nurses (individual) delivering care to patients alongside colleagues (interpersonal), embedded within an ED or larger hospital context (systems).

### Statistical Analysis

Analyses were conducted from April to June 2023. Nurse open-text responses were exported using Stata software, version 17 (StataCorp LLC) into Dedoose software, version 9.2^13^ for data storage, management, and analysis. An initial review of the data was performed by the study authors to identify 1-word responses to exclude for data analysis (n = 1). Rigor and trustworthiness^14,15,16^ were addressed through author reflection about prior assumptions, maintaining an audit log of analytic decisions, and iterative review of codes and categories. A process of reflexivity^17^ was upheld throughout the study. Specifically, the authors intentionally developed a study team with diverse perspectives on the topic of ED nurse work environments as insiders and outsiders to the study context. Two study authors are current or former ED clinicians, and 2 work outside this setting; 3 authors are registered nurses (K.J.M., K.B.L., and J.M.B.C.) and 1 is a physician (R.M.M.).

The authors subsequentially divided the data equally and open coded^18,19^ the text responses using inductive and deductive^20^ (informed by the individual, interpersonal, and systems domains of the Social Ecological Model)^10,11,12^ approaches to identify key concepts in the data for codebook development.^18,21,22^ The codebook included convergent and divergent exemplar quotes for each code. A second round of coding was then conducted by the study authors to condense similar codes or remove codes that did not represent emerging patterns in the open-text responses. The authors assessed a subset of 29 responses (20.4%) for interrater reliability^22^ and reached consensus in the data analytic process (κ = 80). Coding disagreements were adjudicated through a consensus approach in which coauthors discussed coding discrepancies and associated exemplar quotations until a joint decision was reached. Study codes were organized into categories for which the study authors noted (in the coding process)^22,23^ whether a category was a driver of nurses not recommending their workplace or a consequence and what level of the Social Ecological Model was represented. Analytic meetings were held over multiple months to review a written description of the categories, associated codes, and exemplar quotations. The authors condensed categories with similar descriptions and exemplar quotations into larger themes while removing categories that did not address the larger study question or the guiding conceptual model. The coding of the data continued until study categories were refined into larger themes that conveyed why nurses did not recommend their workplace. No new emerging themes in the analytic process signaled data saturation.^14,15,16,17^

## Results

In this qualitative study of 142 emergency nurses (mean [SD] age, 43.5 [12.5] years; 113 [79.6%] female and 28 [19.7%] male; 109 [76.7%] White; 133 [93.7%] non-Hispanic; mean [SD] experience, 14.0 [12.2] years), 94 (66.2%) were licensed to work in New York and the other 48 (33.8%) in Illinois (Table 1). Five themes with associated subthemes were identified from the directed content analysis of open-ended responses (N = 142): unlimited patients with limited support (theme 1), unanswered calls for help (theme 2), license always on the line (theme 3), multidimensional workplace violence (theme 4), and undervalued and unfulfilled (theme 5). Study themes related to a range of social ecological contexts, including individual nurse experiences; their interactions with patients, colleagues, and management; and larger leadership systemic decision-making. Study theme and subthemes are described with contextualized exemplar quotations as follows, with additional quotes in Table 2.

**Table 1.** Characteristics of the Emergency Nurse Sample^a^

Characteristic	Respondents (N = 142)
Age, mean (SD), y	43.5 (12.5)
Sex^b^	
Female	113 (79.6)
Male	28 (19.7)
BSN degree or higher	99 (69.7)
Experience, mean (SD), y	14.7 (12.2)
White race^c^	109 (76.7)
Non-Hispanic ethnicity^d^	133 (93.7)
Recommend place of employment as a good place to work	
Probably not	99 (69.7)
Definitely not	43 (30.3)
State of employment	
Illinois	48 (33.8)
New York	94 (66.2)

Abbreviation: BSN, bachelor of science in nursing.

^a^ Data are presented as number (percentage) of nurses unless otherwise indicated.

^b^ Sex categories included female, male, and other, but the numbers for the other category could not be reported in the table due to small cell size.

^c^ Race categories also included American Indian or Native Hawaiian, Asian, Black or African American, multiracial, or other (no additional information available), but these numbers could not be reported in the table due to small cell sizes.

^d^ Ethnicity categories also included Hispanic, but this could not be reported due to small cell size.

**Table 2.** Themes Explaining Why Emergency Nurses Do Not Recommend Their Workplace

Theme and subtheme	Exemplar quotation [participant No.]
**Theme 1: unlimited patients with limited support**	
Subthemes	
Work environment	“We work short staffed every shift! The acuity of the patients along with no staff and no ancillary staff to help make it a terrible working environment and are major safety concerns for patients and the nurses. I would not recommend anyone to become a nurse or work there or any hospital for that matter! I plan to leave the bedside in the near future!” [RN 104]
	“Staffing is chronically short, dangerous, and exploitative. Admin keeps closing units and consolidating care, leaving the ED overcrowded with boarders. According to many peer reviewed studies, both short staffing and boarding is extremely hazardous, and demeaning, to patients.” [RN 2]
	“I would not recommend my hospital because of low staffing that we are facing all the time. It seems to that no one is addressing the issue and as a result we over worked and not to mention under paid.” [RN 46]
No support staff	“One RN one Doctor and maybe one float for 5+ patients. RN is secretary. Takes all calls makes all calls, ambulates, turns and cleans patients and rooms after discharge. A lot of responsibility for one person.” [RN 17]
	“Not enough RN and ancillary staff to adequately [cover] high census days.” [RN 120]
	“No ancillary staff to assist with EKGs, Labs, CNA duties.” [RN 13]
**Theme 2: unanswered calls for help**	
Subthemes	
Lack of management response	“I do not feel that our concerns as nurses are addressed. I do not feel supported by administration.” [RN 13]
	“Administrative heavy, not concerned with staff, too many travelers, they don’t care about current staff.” [RN 18]
	“Little communication between management and staff nurses. Emails left unanswered and no guidance when we report problems with patient or staff safety issues. I was nearly attacked by a psych patient in a negative pressure room. I was unable to get out easily due to the zippers. I informed my manager via email (it was on night shift) and suggested that for that particular room we switch to plastic sheeting with magnetic closures rather than zippers to allow quick escape for staff (the room is in the psych pod of our ER). I never received a reply - either email or in person. It was never acknowledged that my safety was compromised. The majority of the nurses on my unit (and hospital-wide from what I can tell through talking with other units) feel management would actually not care if we were seriously injured or died due to lack of safety features in place.” [RN 102]
Management discordance	“Not enough staff to care for patients. The patients deserve better. Burn out is [rampant] throughout all disciplines. Upper management appears to have blinders on to this situation.” [RN 24]
	“Healthcare has become too concerned with administrative checkbooks than actually taking care of patients.” [RN 44]
	“Bad staffing, bad compensation, not patient focused.” [RN 9]
	“Management refuses to listen to the nurses concerns for patient safety as patient ratios continue to increase. Administration refuses to admit that we are in a staffing crisis and tries to silence anyone who says we are. There is no praise when you do something good, the focus is always on what you did wrong.” [RN 37]
	“Facility is in transition to different ownership, current management is not focused on safe care. Right now focus is on money not anything else.” [RN 122]
**Theme 3: license always on the line**	
Subthemes	
Compromised care quality	“ER nurses can have up to 17 patients needing varied levels of care including several ICU or stepdown patients with acute life threatening conditions while simultaneously caring for patients who are also ill just not as severe. This is dangerous for the patients and the licenses of the doctors and nurses caring for them.” [RN 2]
	“License always on the line. Critical care patients being held in the ED for days because of lack of competent critical care nurses in the intensive care unit (no fault of the nurses. Fired seasoned nurses, hire new grads to run unit to save money). [Intubated] [patient] held in ED for 8 days waiting to transfer to university hospital. In addition to having this patient nurses had an additional 3 revolving ED rooms, with patients of various acuities. Patients held in ED due to low or almost no staffing. [Patient] returned to ED from surgery because no one to recover patient. A real jungle. No breaks or lunches ever!!” [RN 20]
	“Prior to the pandemic staffing was poor nurse to patient ratios was terrible and my license is at risk everyday during the pandemic it became worse. No one cares about the safety of the nurses no hazard pay no time off dangerous nurse to patient ratios absolutely disgusting what nurses went through and continue to go through especially during the covid pandemic. There is absolutely no respect for nurses the least they could have done was [provide] hazard pay for the hell that we continue to work through. Nursing has become one of the most unsafe terrible paying jobs and [nurses] are treated and continue to be treated like we are not humans.” [RN 29]
	“The lack of staff and lack of supervision will compromise your safety, health and license.” [RN 6]
	“We are too short staffed and put our licenses at risk working in overcrowded understaffed conditions. We also get paid significantly less than surrounding hospitals.” [RN 75]
	“Everyone is doing the best they can but staff to [patient] ratios are consistently unsafe. You are constantly forced to put your license at risk. Staff is burnt out.” [RN 132]
	“Currently, our unit has severe staffing problems. Your license is always at risk.” [RN 141]
Inadequate job protections	“Severely understaffed with a lack of resources and extreme infection control concerns.” [RN 85]
	“…and then a lot of our protocols are [run] by administration decision rather than viewing cases where it may not be appropriate. I am choosing to leave the bedside and pursue becoming a doctor instead.” [RN 53]
**Theme 4: multidimensional workplace violence**	
Subthemes	
Patient	“Patients are rude, entitled, nasty.” [RN 82]
	“Patients are increasingly violent lately and there is no additional security.” [RN 31]
	“...also the escalating violence of patients and visitors in the ER makes it dangerous just to work.” [RN 11]
Colleagues	“‘Experienced’ staff frequently rude and aggressive when offered help with critically ill patients.” [RN 59]
	“[The workplace]…has a strong clique of long-term staff.” [RN 126]
System	“I am speaking to ER. Very high acuity patients into the ER, very low staff (80+ beds and operate at most 24-36), administration does not listen to [its] staff. Has gotten rid of staff and physicians for reasons that are not credible (considering bad working situations, they’ve removed people for speaking out due to poor working situations). Will assign 2+ ICU patients to one nurse with minimal help, while the patients are still unstable. Constantly asking nurses to work [overtime] and burning its staff out. I know multiple people that have applied for positions and are turned down, do not know why considering the ER desperately needs more staff. When I speak with management, they claim they do not see the applications.” [RN 57]
	“When nurses write up unsafe staffing forms to report to the union, they are harassed by managers.” [RN 2]
	“Those that resigned, did so due to lack of support, favoritism that neglected the needs of everyone, and also some to pursue a new position in other departments.” [RN 118]
**Theme 5: undervalued and unfulfilled**	
Subthemes	
Appreciation and respect	“Lack of staff appreciation, lack of positive feedback, very short staffed.” [RN 52]
	“They’re offering huge incentives for nurses already working full time to pick up more time on desperate units but it seems like if the facility would just offer a competitive and [livable] wage staffing could be retained.” [RN 4]
	“The hospital as a whole does not care about the staff they are only worried about patient satisfaction. While that is something that is important I think that staff satisfaction is even more important. The staff have to like their work environment before they can put more effort into helping patients. Administration doesn’t care about nurses.” [RN 124]
	“People are driven into the ground with the workload, responsibility, liability and treated like a dartboard from the public whom expects drive-through service.” [RN 89]
	“Nothing changes and we are underpaid and over worked.” [RN 116]
	“There are not enough staff nurses to be able to run the department safely; administration has brought in agency nurses who are on contract, but have yet to fill the staff positions that were left vacant.” [RN 118]
	“Nurses are not treated with respect, pay is great but not worth the trouble.” [RN 135]
Failing patients	“Understaffing that makes it uncomfortable to get tasks done and to be empathetic and caring.” [RN 91]
	“The nurse to patient ratio is 1:6 in the ER, regardless of acuity and including ICU holds. I leave work feeling frustrated and disappointed that I was unable to provide better care for my patients and their families. I feel like I manage tasks and don’t have time for anything else.” [RN 60]
	“Work is very stressful everyday and we never have enough for patient load or acuity.” [RN 137]

Abbreviations: CNA, certified nursing assistant; ED, emergency department; EKG, electrocardiogram; ER, emergency room; ICU, intensive care unit; RN, registered nurse.

### Theme 1: Unlimited Patients With Limited Support

#### Work Environment

Although emergency nurses described their work environment as inherently accommodating of high patient workloads, these conditions were worsened by insufficient staffing, inadequate specialty nurse training, and prolonged patient boarding. As one nurse stated, “The ED requires nurses to take an unlimited amount of patients. I have had 12 patients including ICU [intensive care unit] and stepdown holds at the same time. The ER [emergency room] is full of boarding patients who receive suboptimal care. There is no dedicated resuscitation/trauma nurse.”

#### No Support Staff

In addition to inadequate nurse staffing, the staffing of unlicensed assistive personnel (ie, patient care technicians and nursing assistants) was poor and significantly hampered nurses’ ability to deliver emergency care. The shortage of ancillary staff deterred important care interventions, such as vital signs, transporting patients, and 1-on-1 monitoring of patients. A nurse stated, “ED nurses are required to manage 4 patients and there is almost no added ancillary help except for 1 tech who often gets put on a patient watch. The amount of work is nearly unmanageable and is verging on the [edge of] unsafe.”

### Theme 2: Unanswered Calls for Help

#### Lack of Management Response

A contributor to nurses not recommending their workplace was a lack of unit leadership response to nurse reports on unsafe working conditions and requests for help. Nurses specifically described administrators’ lack of responsiveness to inadequate unit staffing and incidents of patient violence in the ED. One nurse described, “Little communication between management and staff nurses. Emails left unanswered and no guidance when we report problems with patient or staff safety issues.”

#### Management Discordance

Nurses reported that a lack of manager responsiveness to reports of unsafe working conditions was attributed in part to a disconnect between nurse and leadership priorities. Nurses identified that administration decision-making and actions were targeted more toward health care financials as opposed to patient care and work environment safety: “[Administration] does not care about the patients, all about the money. Cutting staff to dangerous levels so upper management gets bonuses to cut the budget.”

### Theme 3: License Always on the Line

#### Compromised Care Quality

Unsafe working conditions exacerbated poor-quality care delivery that compromised patient outcomes and increased nurses’ fears of losing their licenses. The culture of continually unsafe working conditions contributed to nurses’ perception that their license was on the line. Nurses used this metaphor to describe a sense of helplessness and fear that they would be affiliated with care that endangered patients: “Upper management does not care or listen to the staff. Our ratios are unsafe and our staffing is unsafe. I feel that my nursing license is in jeopardy every time I go to work.”

#### Inadequate Job Protections

Nurses’ concerns about their licenses being in jeopardy were driven in part by a lack of infrastructure for ensuring that high-quality care was delivered. Nurses described inadequate infection control protocols, equipment, and safety regulation transparency in their work environment: “…poor staff morale, lack of transparency from administration re: changing work regulations, job security and future, sloppy infection control management, aging disrepair of building and equipment, frequent daily nursing staff call-ins, shortages of equipment supplies specific to patient care routines and diagnostics, etc.”

### Theme 4: Multidimensional Workplace Violence

#### Patient

As first-line clinicians for patients entering the hospital, emergency nurses reported physical and verbal workplace violence from patients. Workplace violence from patients was reported as increasing and exacerbated by a lack of support resources, such as safe staffing and security. As one nurse described, “Nurse safety comes second to patient satisfaction. Triage nurse is expected to sit directly in front of the door with security behind them near the back of the room. Obviously not the safest set up. Nurses have been verbally and physically assaulted out there, yet nothing has changed.”

#### Colleagues

Workplace violence in the form of bullying across levels of clinician tenure (ie, years of experience) and position type (ie, contract vs permanent nurse) contributed to nurses not recommending their workplace. One nurse described a “bully environment that chases out new nurses.” Another nurse stated, “I am a traveler. Staff is brutal to travelers. Attitudes are poor.”

#### System

Systemic, cultural drivers of workplace violence were reported by nurses in the form of discrimination and racism, management threats of retribution, and reported favoritism of management toward nursing staff. As one nurse said, “Those that resigned did so due to lack of support, favoritism that neglected the needs of everyone, and also some to pursue a new position in other departments.”

### Theme 5: Undervalued and Unfulfilled

#### Appreciation and Respect

Nurses reported feeling undervalued by hospital management when they continued to work in continually unsafe working conditions. Nurses reported a lack of appreciation from unit management, patients, and the larger hospital system: “The hospital system does not place value on retaining nurses. There is very little incentive for working there year after year. Raises are a pittance and are insulting to years of service. New grads are coming in making more pay than nurses with 8+ years of experience.”

#### Failing Patients

A key factor in nurses not recommending their workplace was a lack of fulfillment working in unsafe environments where optimal patient care delivery was compromised daily. Nurses reported a sense of disappointment and frustration leaving their jobs with tasks left undone and suboptimal care delivered to patients. Nurses endorsed wages as a contributing factor to remain in their job, however at a cost of professional and personal fulfillment. One nurse said, “The only reason I am there is the money, which is at the cost of my happiness and it is becoming more and more apparent that I would rather be happy with $1750 paychecks than unhappy with $3400 paychecks.”

## Discussion

Emergency nurses did not recommend their hospital ED as a good place to work due to insufficient nurse staffing, a lack of management responsiveness, unsafe working conditions, workplace violence, and perceptions of being undervalued. Nurses’ concerns that their licenses were in jeopardy were driven by a reported lack of leadership investment in adequate staffing, infrastructure (ie, equipment), and care protocols that ensured high-quality and safe patient care. Nurses felt undervalued in their jobs and unfulfilled in providing suboptimal care for patients, which could be improved with better leadership investment in nurse staffing. Our results, contextualized within the Social Ecological Model (Box), demonstrate that nurses do not recommend their workplace due to reported systemic organizational inadequacies (eg, long-term nurse understaffing) that manifest at the interpersonal level with patients (eg, workplace violence) and hospital leadership (eg, lack of management responsiveness).

Box. **Study Themes Across Social Ecological Model Level**

**System (Themes 1,^a^ 2,^b^ 3,^c^ and 4^d^)**

Unfavorable work environments
Poor nurse and ancillary support staffing
Lack of management responsiveness
Retribution for reporting unsafe working conditions
Inadequate job protections (eg, infection control)

**Interpersonal (Themes 1 and 4)**

Patient-to-nurse verbal and physical assault
Colleague workplace bullying

**Individual Nurse (Themes 1, 2, 3, and 5^e^)**

Nurse perception that license is at risk
Undervalued by management
Unfulfilled delivering suboptimal patient care

^a^
Theme 1: unlimited patients with limited support.

^b^
Theme 2: unanswered calls for help.

^c^
Theme 3: license always on the line.

^d^
Theme 4: multidimensional workplace violence.

^e^
Theme 5: undervalued and unfulfilled.

Emergency nurses in our study described the implications of long-term understaffing of nurses and ancillary staff (eg, patient care technicians), suggesting that nurses are important informants to the clinical work environment and the emergency medicine team. Although evidence demonstrates that poor nurse staffing is a driver of poor nurse job outcomes (ie, turnover intention) across many health care units,^24,25,26^ this issue may be heightened in EDs, where patient volumes are variable throughout an entire shift. More than 2 decades of evidence^27,28,29,30^ demonstrates a relationship between hospital nurse staffing and nurse job outcomes (eg, turnover intention), but these data are lacking in the ED context. More evidence is needed to evaluate the relationship between ED staffing and patient and nurse job outcomes.

Another important implication of this study is that nurses did not recommend their workplace to other clinicians if they had unresponsive management and working conditions that inadequately protected them (license always on the line) to care for patients and themselves (infection control). This reality is of grave concern to employers struggling to recruit and retain nurses in their organizations.^4,31,32,33^ Employer investments in management accountability, transparency, and communication are needed for clinicians to be valued and supported in their high-stakes work. Nurses provide concerns regarding the lack of safety in their work settings that compromises patient care outcomes, which could be resolved by ensuring nurses are adequately staffed in their health care units. Most nurses’ safety concerns for patients and themselves were related to nurses’ reports of insufficient staffing in our study.

Emergency nurses cited workplace violence across multiple domains (eg, interpersonal and systemic), thus highlighting the need for better incident reporting and policies. Although emergency clinicians are exposed to the highest rates of physical and verbal assaults in their workplace,^34^ the responsibility to ensure safety from violence must be established at the hospital employer level.^35^ Evidence demonstrates that employer investments in the features of high-quality work environments^31^ (eg, effective hospital management, foundations for safety, and safe nurse staffing) are associated with better nurse safety reports^1^ and are a strategy to address workplace violence.

An important final implication of this study is the lack of fulfillment that nurses reported in the inability to provide high-quality, safe patient care in the ED. The unsafe working conditions reported by nurses in this study suggest a degrading of nurses’ intent to remain in their job when left with minimal resources at work and a lack of support from upper management. Even when working in well-compensated (ie, wage) positions, emergency nurses reported that unsafe working conditions compromised their well-being and confidence that patients received safe, high-quality care. These findings are supported by existing evidence demonstrating that investments in better work environments and safe staffing have a greater influence on nurses’ burnout and turnover intentions than wages.^36^ Although improving patient outcomes in the ED is a key priority for clinicians, it is important to emphasize that nurses’ motivation to remain in their jobs depends on their work environment supports.^1,31^ In other words, hospitals’ failure to invest in safe working conditions may degrade or dehumanize nurses’ motivation to improve outcomes for patients, which may contribute to the persistent cycle of nurse burnout and turnover documented across the health services nursing literature.^1,25,37^

### Limitations

This study has some limitations. The COVID-19 pandemic is an important contextual feature that may have influenced nurses’ reports on their work environment; however, a remarkable finding was that most nurse responses were not directly related to pandemic-related issues. These findings align with more than 2 decades of evidence, including data just before the pandemic,^3,38^ demonstrating that hospitals have continually understaffed health care units, exposing patients and nurses to unsafe working conditions. Recent evidence from nurses who left health care between 2018 and 2021 demonstrates that the leading factors contributing to employment departures were not pandemic specific: planned retirement, burnout, and insufficient staffing.^39^ Nurses in our study were prompted to provide an open-text response if they probably or definitely did not recommend their workplace to other clinicians. Thus, we do not know the counterfactual to what motivates nurses to recommend their place of employment. However, that 40.7% of emergency nurses would not recommend their workplace in the context of high emergency clinician burnout and turnover critically informed our motivation to explore this line of inquiry related to the emergency workforce. Finally, we do not have data on hospital characteristics, including union status, to evaluate variations in nurse recommendations of their workplace; however, the issue of nurse recruitment and workplace dissatisfaction is salient across all types of hospitals.

## Conclusions

In this qualitative study of open-text responses, nurses did not recommend their workplace as a good place to work because of poor nurse and ancillary staffing, nonresponsive hospital leadership, unsafe working conditions, workplace violence, and a lack of feeling valued. To transform EDs to attractive workplaces for clinicians, health care administrators should focus on improving nurse staffing and communication between nurses and hospital management and ensuring an adequate sense of nurse value as a foundation to mitigate poor patient quality and safety outcomes and workplace violence.

## Notes

Supplement.
Data Sharing Statement
