# Supplementary material for: Emergency Nurses’ Reasons for Not Recommending Their Hospital to Clinicians as a Good Place to Work
Source: JAMA Netw Open. 2024 Apr 9;7(4):e244087. doi: 10.1001/jamanetworkopen.2024.4087 (PMC11004828; doi:10.1001/jamanetworkopen.2024.4087)
Supplement: Supplement. — Data Sharing Statement [file jamanetwopen-e244087-s001.pdf]

Data Sharing Statement

Muir. Emergency Nurses' Reasons for Not Recommending Their Hospital to Clinicians as a Good Place to Work. *JAMA Netw Open*. Published April 09, 2024.
doi:10.1001/jamanetworkopen.2024.4087

Data
Data available: No

Additional Information
Explanation for why data not available: No data to be shared
